# Matured Hop Bitter Acids in Beer Improve Lipopolysaccharide-Induced Depression-Like Behavior

**DOI:** 10.3389/fnins.2019.00041

**Published:** 2019-01-28

**Authors:** Takafumi Fukuda, Rena Ohya, Keiko Kobayashi, Yasuhisa Ano

**Affiliations:** Research Laboratories for Health Science and Food Technologies, Kirin Company, Ltd., Yokohama, Japan

**Keywords:** matured hop bitter acids, depression, inflammation, vagus nerve, norepinephrine, dendritic spine, hippocampus, brain-gut axis

## Abstract

Recent studies have demonstrated a close association between neural inflammation and development of mental illnesses, such as depression. Clinical trials have reported that treatment with non-steroidal anti-inflammatory drugs is associated with reduced risk of depression. Moreover, nutritional approaches for the prevention and management of depression have garnered significant attention in recent years. We have previously demonstrated that iso-α-acids (IAAs)—the bitter components in beer—suppress hippocampal microglial inflammation, thereby improving cognitive decline. However, effects of hop-derived components other than IAAs on inflammation have not been elucidated. In the present study, we demonstrated that consumption of matured hop bitter acids (MHBAs) generated from α- and β-acids, which show a high similarity with the chemical structure of IAAs, suppress lipopolysaccharide (LPS)-induced cytokine productions in the brain. MHBAs administration increased norepinephrine (NE) secretion and reduced immobility time which represents depression-like behavior in the tail suspension test. Moreover, MHBAs components, including hydroxyallohumulinones and hydroxyalloisohumulones, reduced LPS-induced immobility time. Although further researches are needed to clarify the underlying mechanisms, these findings suggest that MHBAs reduce inflammatory cytokine productions and increase NE secretion, thereby improving depression-like behavior. Similarly, inoculation with LPS induced loss of dendritic spines, which was improved upon MHBAs administration. Additionally, vagotomized mice showed attenuated improvement of immobility time, increase in NE level, and improvement of dendrite spine density following MHBAs administration. Therefore, MHBAs activate the vagus nerve and suppress neuronal damage and depression-like behavior induced by inflammation.

## Introduction

The incidence of mental illnesses, particularly depression, has increased in recent years, which has become a major social problem. The World Health Organization (WHO) has reported that over 300 million people worldwide suffered from depression in 2015. Medical costs related to mental illnesses represent an economic burden, and according to the Organization for Economic Co-operation and Development, they can account for up to 4% of the gross domestic product. Unfortunately, the existing anti-depressant therapies show therapeutic efficacy in as few as one-third of the patients suffering from depressive disorders (Trivedi et al., 2006). Thus, effective treatment for depression remains an unmet clinical need. Recent studies have shown that brain inflammation plays a role in the pathophysiology of major depressive disorders (Dantzer et al., 2008; Hashimoto, 2015; Setiawan et al., 2015; Miller and Raison, 2016). Moreover, anti-inflammatory agents, such as celecoxib and minocycline, decrease depressive symptoms (Kohler et al., 2014; Dean et al., 2017; Husain et al., 2017). Microglia—one of the sites of pro-inflammatory cytokine production—are the key players in the neuronal immune system and are most enriched in the hippocampus. They play critical roles in the development of depression-like phenotype in rodents (Lawson et al., 1990; Zhang et al., 2014). Brain imaging has revealed that microglia in patients suffering from depression are more activated than in healthy subjects (Setiawan et al., 2015). Therefore, anti-inflammatory treatments may be applicable as anti-depressant treatments.

WHO has reported that the proportion of patients suffering from depression who receive appropriate diagnosis and treatment is <10%, particularly in developing countries (World Health Organization, 2017). Nutritional intervention in daily life and nutrition-based approaches for the prevention and management of depression have garnered much attention globally. Recent studies have shown that some nutrients suppress inflammation, leading to the prevention of depression and depression-like behavior. Quercetin (a type of flavonoid) prevented chronic unpredictable stress-induced behavioral dysfunction in mice by alleviating hippocampal oxidative and inflammatory stress (Mehta et al., 2017). Previously, we have demonstrated that iso-α-acids (IAAs)—the hop-derived bitter components in beer—prevented hippocampal inflammation and cognitive impairment by acting on microglia in a mouse model of Alzheimer’s disease and in high-fat diet-induced obese mice (Ano et al., 2017; Ayabe et al., 2018a). However, effects of hop-derived components other than IAAs on inflammation remain unknown.

Matured hop bitter acids (MHBAs), generated by oxidizing α- and β-acids that comprise the bitter components in beer, show chemical structures similar to IAAs derived from α-acids. We have recently reported that MHBAs administration improved hippocampus-dependent memory in mice through increased norepinephrine (NE) secretion in the hippocampus (Ayabe et al., 2018b); however, effects of MHBAs on brain inflammation and mental disorders were not elucidated in that study.

In the present study, we examined whether MHBAs supplementation suppressed inflammation in the hippocampus and evaluated the ability of MHBAs and their components to prevent inflammation-induced depression-like behavior. To this end, we used the acute neural inflammatory animal model induced by lipopolysaccharide (LPS) that demonstrates depression-like behavior (Frenois et al., 2007; Bay-Richter et al., 2011; Lawson et al., 2013; Chen et al., 2017; Li et al., 2017).

## Materials and Methods

### Materials

Matured hop bitter acids and each of its major components [4′-hydroxyallohumulinones (HAH); 4′-hydroxyalloisohumulones (HAIH); tricyclooxyisohumulones A (TCOIH-A); hulupones; and humulinones] were prepared from hop pellets as described elsewhere (Taniguchi et al., 2015a,b). NE was purchased from Sigma-Aldrich Co. (St. Louis, MO, United States) for the analysis of monoamines.

### Animals

Five-week-old male Crlj:CD1 (ICR) mice and vagotomized male ICR mice were purchased from Charles River Laboratories Japan, Inc. (Tokyo, Japan). In the vagotomized mice, vagus nerves were cut under the diaphragm. Vagotomy was performed on 5-week-old ICR mice at Charles River Laboratories Japan, Inc. The control group underwent sham operation. The vagotomized and sham operated mice were used for experimental procedures only after they turned 6–8 week old. Mice were maintained at room temperature (23 ± 1°C) under constant 12/12-h light/dark cycle (light period from 8:00 a.m. to 8:00 p.m.). All mice were acclimatized by feeding a standard rodent diet (CE-2; Clea Japan, Tokyo, Japan) for 7 days before experimental procedures. All animal care and experimental procedures were conducted by July in 2018 in accordance with the guidelines of the Animal Experiment Committee of Kirin Company, Ltd. All efforts were made to minimize animal suffering. All studies were approved by the Animal Experiment Committee of Kirin Company, Ltd.

### Drug Treatment

**FIGURE 1:**
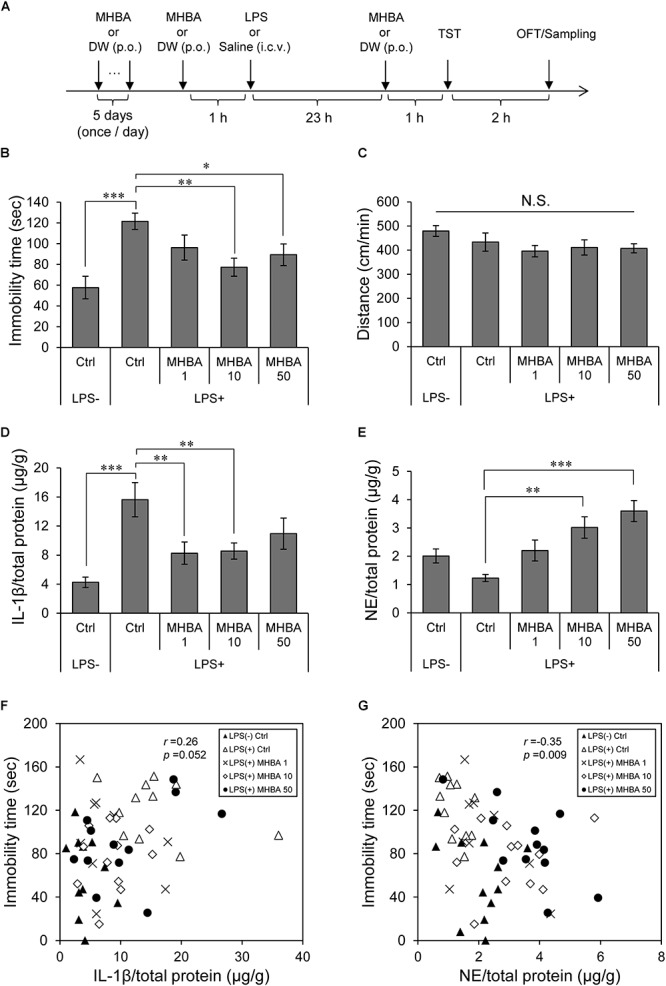
Repeated MHBAs administration improves depression-like behavior induced by LPS and increases norepinephrine (NE) levels in the hippocampus. **(A)** At 6 days after oral treatment with distilled water (DW) or matured hop bitter acids (MHBAs; 1, 10, or 50 mg/kg), saline or lipopolysaccharide (LPS; 15 μg/mouse) was injected intracerebroventricularly to induce depression-like behavior. Additional MHBAs or DW was orally administered 23 h after treatment with LPS or saline. Tail suspension test (TST) was performed 60 min after the final administration of test compounds or DW, and open-field locomotor test (OFT) was performed 120 min after performing TST. **(B)** Immobility time, which represented the depression-like behavior, was evaluated by TST for 6 min. Ctrl indicates control; LPS- and LPS+ indicate the absence and presence of LPS, respectively, and MHBAs 1, 10, and 50 indicate MHBAs concentrations in mg/kg. **(C)** Distance moved in an open field was evaluated for 6 min by OFT following TST. Ctrl indicates control; LPS- and LPS+ indicate the absence and presence of LPS, respectively, and MHBA 1, 10, and 50 indicate MHBAs concentrations in mg/kg. **(D)** Interleukin-1β (IL-1β) levels in hippocampus were measured using ELISA. Ctrl indicates control; LPS- and LPS+ indicate the absence and presence of LPS, respectively, and MHBA 1, 10, and 50 indicate MHBAs concentrations in mg/kg. **(E)** Following OFT, hippocampus samples containing monoamines were prepared. NE levels were determined using HPLC–ECD. Ctrl indicates control; LPS- and LPS+ indicate the absence and presence of LPS, respectively, and MHBA 1, 10, and 50 indicate MHBAs concentrations in mg/kg. **(F)** Correlation between immobility time and hippocampal IL-1β levels is shown (Pearson correlation coefficient *r* = 0.26, *P* = 0.052). **(G)** Correlation between immobility time and hippocampal NE levels is shown (Pearson correlation coefficient *r* = 0.35, *P* = 0.009). All values are expressed as means and SEM (*n* = 9–12 mice per group). Experimental data were analyzed by Dunnet’s test; ^∗^*P* < 0.05, ^∗∗^*P* < 0.01, and ^∗∗∗^*P* < 0.001 compared with LPS-treated group administered DW.

Test compounds [MHBAs; 1, 10, or 50 mg/kg, HAIH; 10 mg/kg, TCOIHA; 1 mg/kg, HAH; 1 mg/kg] or distilled water (DW) were orally administered once a day for 6 consecutive days after habituation. To induce brain inflammation after 60 min of the last administration, mice were deeply anesthetized with sodium pentobarbital (Kyoritsu Seiyaku, Tokyo, Japan), and LPS (15 μg/mouse, from *Escherichia coli* O111:B4; Sigma Aldrich, St. Louis, MO, United States) or saline (for sham-operated controls) was then injected by hand into the cerebral ventricle in a volume of 5 μL/hemisphere in accordance with a previous study (Ano et al., 2015). At 23 h after the injection of LPS or saline (experimental day 7), mice were orally administered with test compounds or DW and subjected to behavioral evaluation (Figure 1A); cytokine and NE content were analyzed. In experiments using vagotomized mice, animals were subjected to Golgi–Cox staining after behavioral evaluation.

### Tail Suspension Test (TST)

Tail suspension test (TST) was performed 60 min after the final administration of test compounds or DW to evaluate the effect of test compounds on depression-like behavior. One end of an adhesive tape (width 1 cm, length 15 cm) was fixed to the upper surface of a box such that the head of the mouse, in an inverted state, would be at a height of 45–50 cm from the bottom of the box. The other end (2 cm) of the tape was firmly wrapped around the tail (1 cm from the tip) and the mouse was suspended from its tail for 6 min. The mice were visually inspected from a separate room using a video camera and monitor, and the immobility time within 6 min was evaluated. Mice were considered immobile only when they hung passively and completely motionless.

### Open-Field Locomotor Test (OFT)

To measure locomotor activities, open-field locomotor test (OFT) was performed 120 min after TST. Mice were individually placed at the centre of a cubic chamber (40 cm × 40 cm × 40 cm) made of gray polyvinyl chloride. Horizontal movements of mice were measured using automatic actography (SMART Video Tracking System, Harvard Apparatus, Holliston, MA, United States). The test lasted for 6 min.

### Brain Sample Preparation for Measuring of Cytokine and NE Levels

The hippocampus was collected from each hemisphere and homogenized using a multi-bead shocker (Yasui Kikai, Osaka, Japan) in RIPA buffer (Wako, Osaka, Japan) containing a protease inhibitor cocktail (BioVision, Mountain View, CA, United States). Supernatants were collected after centrifugation at 14,000 rpm for 30 min.

### Analysis of Inflammation in the Hippocampus

Supernatant obtained after processing of the left hippocampus was used for cytokine evaluation. Total protein concentration of each supernatant was measured using the BCA Protein Assay Kit (Thermo-Scientific, Yokohama, Japan). For quantifying cytokines, supernatant was evaluated using enzyme-linked immunosorbent assay (ELISA) kit (eBiosciences, San Diego, CA, United States) in accordance with manufacturer’s instructions.

### Analysis of NE in the Hippocampus

Supernatant obtained after the processing of the right hippocampus was used for monoamine analysis after treatment with 0.2 M perchloric acid and filtration using 0.22 μM membrane filter (Millipore, Bedford, MA, United States). Monoamines were quantified using high-performance liquid chromatography coupled with electrochemical detection (HPLC–ECD; Eicom, Kyoto, Japan) using Eicompak SC-5ODS and PrePak columns (Eicom, Kyoto, Japan). The mobile phase comprised 83% 0.1 M acetic acid in citric acid buffer (pH 3.5), 17% methanol (Wako, Osaka, Japan), 190 mg/mL of sodium 1-octanesulfonate sodium (Wako, Osaka, Japan), and 5 mg/mL EDTA-Na_2_. For ECD, the applied voltage was 750 mV vs. an Ag/AgCl reference electrode.

### Golgi–Cox Staining and Evaluation of Dendritic Spine Density

To evaluate dendritic spine density in the hippocampus, the left hemispheres were stained using commercially available FD Rapid GolgiStain Kit (FD Neurotechnologies, Columbia, MD, United States) following manufacturer’s instructions. Brains were immersed in the impregnation solution, which comprised equal volumes of Solutions A and B, and stored at room temperature for 2 weeks in the dark. The impregnation solution was replaced with fresh solution after the first 6 h of immersion. The brains were then transferred into Solution C and stored at room temperature for 72 h in the dark. The solution was replaced with fresh solution on the next day. Stained brains were cryosectioned at -2.16 mm relative to Bregma in horizontal section planes at a thickness of 100 μm. Hippocampal CA3 dendrites were microscopically imaged using a UPlanApo 20× objective (Olympus, Tokyo, Japan), and spines were counted starting from their point of origin from the primary dendrite to the secondary branch of dendrites. For the measurement of spine densities, only those spines that emerged perpendicular to the dendritic shaft were counted. Regions of 20–30 μm were randomly and individually selected from all the neurons where the image was clearly captured, and spine density was evaluated. Consequentially, three to five neurons were measured per mouse, and average value was calculated as the individual value for the mouse.

### Statistical Analysis

All values are expressed as means and SEM. Experimental data were analyzed by Dunnett’s test or Tukey–Kramer’s test. *P* < 0.05 was considered statistically significant. All statistical analyses were performed using the BellCurve for Excel (Social Survey Research Information Co., Ltd., Tokyo, Japan).

## Results

### Effects of MHBAs on LPS-Induced Depressive Behavior

To evaluate effects of MHBAs on depression-like behavior induced by inflammation, we conducted TST using mice that were intracerebroventricularly inoculated with LPS. TST test revealed that immobility time in LPS-treated mice was significantly longer than that in sham-treated mice, indicating LPS-induced depression-like behavior in mice (Figure 1B). In contrast, the immobility time in LPS-inoculated mice treated with MHBAs at 10 or 50 mg/kg was significantly shorter than that in LPS-inoculated sham-treated mice. Locomotor activities, as evaluated by OFT, did not differ among the groups (Figure 1C). These results indicate that MHBAs improved LPS-induced depression-like behavior. Moreover, we measured interleukin (IL)-1β levels in the hippocampus to evaluate inflammation level (Figure 1D). IL-1β level in the hippocampus of LPS-inoculated mice was significantly increased compared with that in the hippocampus of sham-treated mice but significantly decreased in the hippocampus of LPS-inoculated mice treated with either 1 or 10 mg/kg MHBAs. These results suggest that MHBAs prevent inflammation in the hippocampus. Previous studies have shown that NE might play important roles in depression-like behavior (Liu et al., 2012; Barua et al., 2018). Thus, we quantified NE level in the hippocampus of mice using HPLC (Figure 1E). NE levels in the hippocampus of mice treated with 10 and 50 mg/kg MHBAs were significantly increased. IL-1β (*r* = 0.26, *P* = 0.052) (Figure 1F) and NE (*r* = -0.35, *P* = 0.009) (Figure 1G) levels in the hippocampus of mice were weakly correlated with immobility time observed in TST.

### Effects of Major Compounds in MHBAs on LPS-Induced Depression-Like Behavior

**FIGURE 2:**
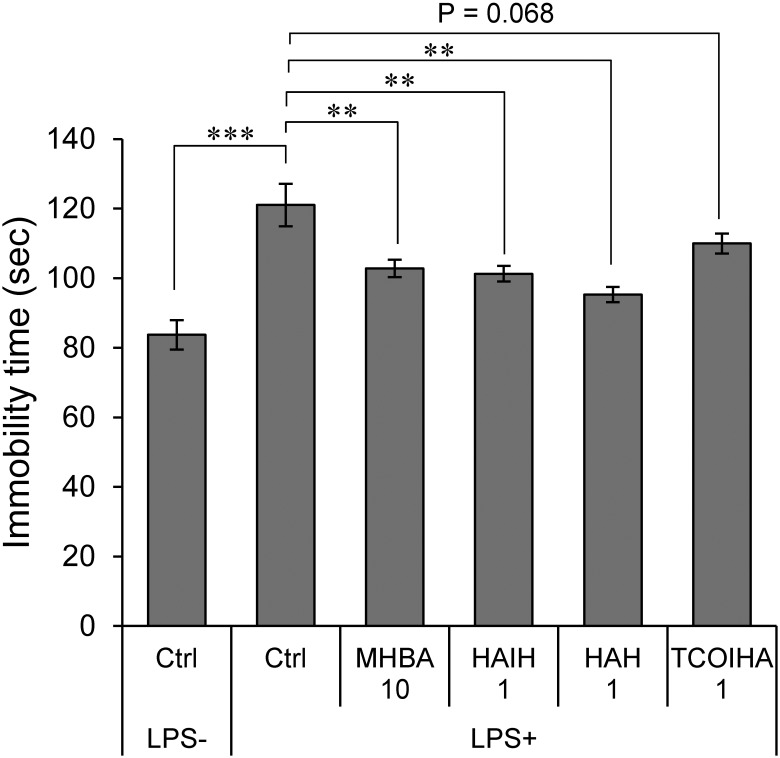
Major compounds in MHBAs exert anti-depressant effect. Effects of MHBAs (10 mg/kg) and each of its major compounds [HAIH (1 mg/kg), HAH (1 mg/kg), and TCOIHA (1 mg/kg)] on depression-like behavior were evaluated with TST using an identical procedure reported in Figure 1A. Ctrl indicates DW-treated control group; LPS- and LPS+ indicate the absence and presence of LPS, respectively and MHBA 10, and HAIH, HAH, and TCOIHA indicate concentrations of each compound in mg/kg. All values are expressed as means and SEM (*n* = 5 mice per group). Experimental data were analyzed by Dunnet’s test; ^∗∗^*P* < 0.01 and ^∗∗∗^*P* < 0.001 compared with LPS-treated group administered DW.

Matured hop bitter acids constitute both α- and β-acid oxidants with β-tricarbonyl structures (Taniguchi et al., 2015a,b). Effects of three major constituents (HAH, HAIH, and TCOIHA) of MHBAs on depression-like behavior were examined in TST. Immobility time in mice treated with 1 mg/kg HAH and HAIH was significantly lower than that in the sham-treated mice and was equivalent to the effect of MHBAs at 10 mg/kg (Figure 2). Moreover, mice treated with 1 mg/kg TCOIHA showed a reduced immobility time (*P* = 0.068). These results indicate that major compounds in MHBAs contribute to the suppression the concomitant depression-like behavior in TST induced by inflammation.

### Involvement of the Vagus Nerve in Effects of MHBAs on Depression-Like Behavior

**FIGURE 3:**
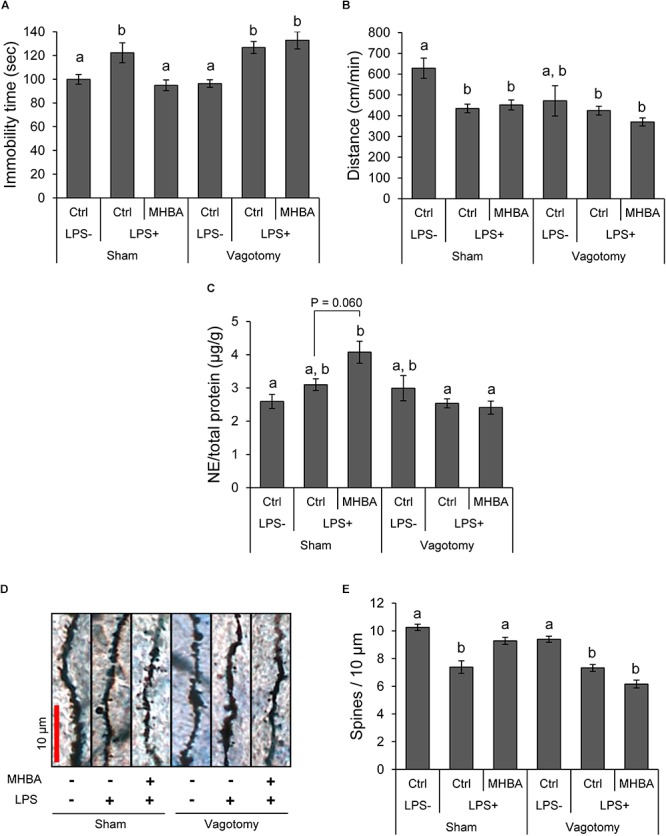
Effects of MHBAs on depression-like behavior were mediated by vagus nerve stimulation. Five-week-old mice are vagotomized and used for experimental procedures at 6–8 weeks of age. The drug treatment procedure was essentially similar to that reported in Figure 1A. **(A)** Anti-depressant effects of MHBAs (10 mg/kg) on sham or vagotomized mice were evaluated using TST. **(B)** Distance moved in open field was evaluated for 6 min using OFT following TST. **(C)** After OFT, mice were sacrificed and hippocampal samples containing monoamines were prepared. Norepinephrine (NE) levels were determined using HPLC–ECD. **(D,E)** Density of dendritic spines in the hippocampus was evaluated by Golgi–Cox staining. Ctrl indicates control, and LPS- and LPS+ indicate the absence and presence of LPS, respectively. Experimental data were analyzed using Tukey–Kramer’s test (*n* = 5–7 mice per group). *P* < 0.05 was considered significant. Different letters indicate significant differences (*P* < 0.05) between the groups.

To elucidate whether MHBAs act through the blood–brain barrier (BBB) or through the vagus nerve, vagotomized or sham-treated mice were administered oral MHBAs and subjected to TST. Immobility time in LPS-inoculated vagotomized mice was significantly longer than that in saline-inoculated vagotomized mice (Figure 3A). In contrast, immobility time remained unchanged in vagotomized mice administered MHBAs or DW. Locomotor activities in OFT remained unchanged among all vagotomized groups (Figure 3B). These results indicate that the vagus nerve was involved in effects of MHBAs on the improvement of LPS-induced depression-like behavior.

In sham mice, NE level in the hippocampus increased upon MHBAs administration [vs. LPS (+) Ctrl, *P* = 0.060] (Figure 3C). However, in vagotomized mice, NE levels did not change upon MHBAs administration. We used Golgi–Cox staining to evaluate effects of MHBAs on dendritic changes in LPS-inoculated mice. The density of dendritic spines in the hippocampus significantly decreased after LPS treatment (Figure 3D,E), which was consistent with previous reports (Zhang et al., 2014; Ma et al., 2017). MHBAs administration improved reduction in the density of dendritic spines in LPS-inoculated mice. Conversely, no such improvement was observed upon MHBAs administration in vagotomized mice. These results suggest that effects of MHBAs on depression-like behavior and hippocampal NE levels are mediated by the vagus nerve.

## Discussion

Inflammation can elicit severe behavioral changes, including the onset of depressive symptoms characterised by feelings of sadness and fatigue and withdrawal of social behavior (Slavich and Irwin, 2014). To the best of our knowledge, the present study is the first report to demonstrate that the administration of MHBAs derived from hops improves LPS-induced depression-like behavior. In addition, elevated NE levels and suppressed IL-1β levels were confirmed. Previous studies have indicated that chronic stress significantly increases hippocampal IL-1β levels (Liu et al., 2014; Zhang et al., 2016), which plays a key role in the development of depression owing to the high density of hippocampal microglia (Bluthe et al., 2000; Tsai, 2017; Yamawaki et al., 2018). In the present study, IL-1β levels in the hippocampus were correlated with immobility time in TST (Figure 1F). Therefore, although further studies are required to prove a direct causal relationship between the attenuation of depression-like behavior and the suppression of inflammatory responses by MHBAs.

Moreover, depression is characterized by dendritic changes (Duman and Duman, 2015). MHBAs administration prevented dendritic loss induced by LPS (Figure 3D,E). Decrease in dendritic spine density due to inflammation is mediated by decrease in levels of neural protective factors, such as BDNF (Zhang et al., 2014). Therefore, anti-inflammatory effects of MHBAs are certainly one of the protective mechanisms against changes in dendritic spine density.

MHBAs are a mixture of α- and β-acid oxidants, which are characterized by a common β-tricarbonyl moiety (Taniguchi et al., 2015a,b). We demonstrated that HAIH and HAH significantly improved LPS-induced depression-like behavior and TCOIHA tended to improve symptoms (Figure 2). Thus, the common β-tricarbonyl moiety of HAIH, HAH, TCOIHA, and IAAs might contribute to the improvement of depression-like behavior.

Consistent with a previous report, our results indicated that MHBAs administration increased NE levels in the hippocampus (Ayabe et al., 2018b). Hippocampal NE levels and immobility time in TST were weakly correlated (Figure 1G). Increased hippocampal NE levels reportedly contribute to the improvement of depression-like behavior (Liu et al., 2012; Barua et al., 2018). This might partly explain the mechanism underlying the suppression of inflammation-induced depression-like behavior in TST by MHBAs. However, decrease in immobility time after MHBAs administration to non-LPS-treated mice was not significant (data not shown), suggesting that the suppression of depression-like behavior in TST was not mediated by NE alone; moreover, the concentration of MHBAs (at 50 mg/kg) that mediated the highest increase in NE level did not induced the shortest immobility time. Previous studies performed under normal conditions have suggested that NE aids microglia in brain function regulation (Kaczmarczyk et al., 2017). Adrenergic β1- and β2-receptors are the only functionally significant adrenergic receptors in the microglia, which are activated primarily by NE (O’Donnell et al., 2012). Thus, increase in NE levels mediated by MHBAs might suppress increase in inflammatory cytokine levels in the microglia. However, since anti-inflammatory effects of MHBAs and NE levels were not significantly correlated (*r* = -0.12, *P* = 0.37, data not shown), the existence of several mechanisms underlying these anti-inflammatory effects are plausible. Further studies are warranted to elucidate the association between the suppression of inflammation and increase in NE production in the hippocampus.

Furthermore, the anti-depressant effects of MHBAs were mediated by the vagus nerve (Figure 3), suggesting that vagus nerve stimulation (VNS) from the intestinal tract is involved in the effect of MHBAs on depression-like behavior rather than the direct absorption of MHBAs into the brain through BBB. In 2005, the Food and Drug Administration has approved VNS therapy using a small device, which elicits electrical stimulation, for managing treatment-resistant depression (Sackeim et al., 2001). Despite its effectiveness in treating depression, the underlying mechanisms through which VNS mediates depression remain unclear (Nemeroff et al., 2006; Vonck et al., 2014). Previous studies have indicated that VNS increases hippocampal NE levels via the locus coeruleus (Hassert et al., 2004; Roosevelt et al., 2006). Taken together, VNS may exert its positive effects against depression by increasing NE levels in the brain and suppressing of inflammation.

## Conclusion

MHBAs administration suppressed neural inflammatory responses, increased hippocampal NE levels, and attenuated LPS-induced depression-like behavior. Furthermore, these effects of MHBAs were mediated by VNS. However, detailed mechanisms underlying anti-depressant effects of MHBAs warrant further research. Activation of the vagus nerves through specific diet, which can in turn improve depression-like behavior, is a safe and novel approach for treating depression.

## Author Contributions

TF and YA designed and performed the experiments, analyzed the data, and wrote the paper. RO and KK performed the experiments.

## Conflict of Interest Statement

All authors are employees of Kirin Company, Ltd.
